# Brain responses to biological motion predict treatment outcome in young children with autism

**DOI:** 10.1038/tp.2016.213

**Published:** 2016-11-15

**Authors:** D Yang, K A Pelphrey, D G Sukhodolsky, M J Crowley, E Dayan, N C Dvornek, A Venkataraman, J Duncan, L Staib, P Ventola

**Affiliations:** 1 Autism and Neurodevelopmental Disorders Institute, The George Washington University and Children's National Health System, Washington, DC, USA; 2 Child Study Center, Yale University School of Medicine, New Haven, CT, USA; 3 Department of Radiology and Biomedical Research Imaging Center, The University of North Carolina at Chapel Hill, Chapel Hill, NC, USA; 4 Department of Electrical and Computer Engineering, Johns Hopkins University, Baltimore, MD, USA; 5 Department of Radiology and Biomedical Imaging, Yale University School of Medicine, New Haven, CT, USA; 6 Department of Biomedical Engineering, Yale University, New Haven, CT, USA

## Abstract

Autism spectrum disorders (ASDs) are common yet complex neurodevelopmental disorders, characterized by social, communication and behavioral deficits. Behavioral interventions have shown favorable results—however, the promise of precision medicine in ASD is hampered by a lack of sensitive, objective neurobiological markers (neurobiomarkers) to identify subgroups of young children likely to respond to specific treatments. Such neurobiomarkers are essential because early childhood provides a sensitive window of opportunity for intervention, while unsuccessful intervention is costly to children, families and society. In young children with ASD, we show that functional magnetic resonance imaging-based stratification neurobiomarkers accurately predict responses to an evidence-based behavioral treatment—pivotal response treatment. Neural predictors were identified in the pretreatment levels of activity in response to biological vs scrambled motion in the neural circuits that support social information processing (superior temporal sulcus, fusiform gyrus, amygdala, inferior parietal cortex and superior parietal lobule) and social motivation/reward (orbitofrontal cortex, insula, putamen, pallidum and ventral striatum). The predictive value of our findings for individual children with ASD was supported by a multivariate pattern analysis with cross validation. Predicting who will respond to a particular treatment for ASD, we believe the current findings mark the very first evidence of prediction/stratification biomarkers in young children with ASD. The implications of the findings are far reaching and should greatly accelerate progress toward more precise and effective treatments for core deficits in ASD.

## Introduction

Within autism spectrum disorders (ASDs), core social communication symptoms are key targets for the development of pharmacological and behavioral interventions.^1^ Recent clinical trials of behavioral interventions report favorable results.^2, 3, 4^ Yet, the promise of targeted, precision medicine^5^ for core social communication deficits in ASD is hindered by a fundamental problem: the lack of sensitive, objective markers to identify subgroups of young children more or less likely to respond to specific treatments. By objectively measuring brain responses, functional neuroimaging techniques provide a promising solution to this problem. These techniques have revealed key neuroanatomical circuits implicated in core ASD deficits, including networks of brain regions engaged in social reward/social motivation^6^ (for example, social orienting, seeking and enjoying social engagements, and maintaining social contact), social attention and action observation^7, 8^ (for example, goal-directed eye-gaze following), and social perception^9, 10^ (for example, face recognition, action perception, emotion decoding). Here, using a well-validated biological motion functional magnetic resonance imaging (fMRI) paradigm^11^ that robustly engages the neural circuits supporting social motivation and social information processing, we sought to identify prediction/stratification biomarkers that can accurately forecast the response to an evidence-based behavioral treatment—pivotal response treatment^2^ (PRT)—in young children with ASD.

We investigated the accuracy of fMRI neurobiomarkers in predicting treatment response in a sample (*N*=20; 7 girls, 13 boys) of young (mean age=5.90 years, s.d.=1.07 years), cognitively able (mean IQ=103.45, s.d.=17.03) children with ASD. These children participated in a 16-week trial of PRT, a behavioral treatment focused on social communication skill development.^2, 12^ The treatment, PRT, is one of a very few evidence-based treatments for children with ASD. It targets pivotal areas, including social initiation and social responsivity with the premise that improvements in these areas lead to more widespread and generalized improvements in multiple domains of development representing core changes in social motivation.^2, 12^ PRT consisted of 16 weeks of treatment, 7 h per week including 5 h per week of direct intervention with the child and 2 h per week of parent guidance. The primary clinical outcome measure was the total raw score from the Social Responsiveness Scale (SRS),^13, 14^ a parent report measure of social functioning. Treatment effectiveness was modeled as the delta change scores (that is, post minus pre) of the SRS total raw scores.

Social orienting and visual sensitivity to biological motion are evolutionarily well conserved, reflecting an ontogenetically early-emerging mechanism, fundamental to adaptive social engagement.^10, 15, 16, 17, 18^ We targeted neuroanatomical networks involved in social motivation and social information processing via a well-validated point-light display biological motion paradigm. During a 5 min fMRI scan at 3 Tesla, conducted at baseline within 1 week before PRT treatment, our study participants viewed neuroimaging stimuli depicting point-light displays of coherent biological (BIO) or scrambled biological (SCRAM) motion, created from motion capture data (that is, videos created by placing lights on the major joints of a person and filming them moving in the dark).^11, 19^

Although relatively impoverished stimuli, point-light displays contain sufficient information to identify the kind of motion being produced (for example, walking, dancing, reaching), as well as the identity of the agent.^20^ Unique sensitivity to point-light displays is present across species and early in postnatal development. For instance, newly hatched chicks recognize biological motion in point-light displays,^15^ and 2-day-old human infants preferentially attend to biological motion in point-light displays.^16^ Importantly, in a group of 2-year-old children with ASD, Klin *et al.*^19^ documented a failure to orient preferentially toward point-light displays of canonical biological motion. Elsewhere, disrupted perceptual sensitivity to biological motion has been documented in older children with ASD.^21^ Our prior neuroimaging work identified dysfunction in the biological motion processing system as reflecting key neural signatures of ASD in affected children and as a neuroendophenotype of genetic risk in unaffected siblings of children with ASD.^11^ Through our biological motion task, the present report leveraged these prior discoveries, targeting the neural systems involved in social motivation and social information processing.

## Materials and methods

### Participants

The study participants included 20 children with a primary diagnosis of ASD (mean age=5.90 years, s.d.=1.07; 7 females, 13 males). Cognitive ability was measured using the DAS-II (Differential Ability Scales-Second Edition).^22^ All the participants were cognitively able (IQ⩾70; range=70–128). All the participants met DSM-5^1^ diagnostic criteria for ASD as determined by expert clinical judgment. This judgment was supported by the results of gold-standard diagnostic instruments—the ADI-R (Autism Diagnostic Interview-Revised)^23^ and ADOS (Autism Diagnostic Observation Schedule)^24^—administered by research-reliable and licensed clinical psychologists. All the participants were free of psychotropic medication. No changes in educational placement or major changes in educational services were reported by the parents while their children were in the study. Pretreatment clinical behavioral measures included (a) the parent-reported SRS^13, 14^ as a continuous, quantitative measure of ASD core symptom severity, (b) the clinician-administered CELF (Clinical Evaluation of Language Fundamentals)^25, 26^ as a measure of core language ability (receptive and expressive) and (c) the clinician-administered Vineland-II (Vineland Adaptive Behavior Scales-Second Edition)^27^ as a measure of adaptive behaviors. Comprehensive demographics and characterization information are provided in Table 1. The study is registered at ClinicalTrials.gov (ID: NCT01908686).

Inclusion criteria for all the participants included being in good medical health and being cooperative with testing; exclusion criteria for all the participants included a history of significant head trauma or serious brain or psychiatric illness, as well as current use of prescription medications that may affect cognitive processes under study (see the ClinicalTrials.gov registry for complete inclusion and exclusion criteria). Two children (beyond the 20 participants) were screened and did not qualify for the study based on the above criteria. One child had significant, uncontrolled seizures, and the other child exhibited very highly disruptive behaviors so was unable to complete the screening assessments. Two other participants (beyond the 20 participants) were not included in the analysis because of missing valid SRS data. All the participants passed MRI safety screening, including being free of any metal implants and evidence of claustrophobia. Written informed consent was obtained from each participant's parent(s), and assent was obtained from each child. The Human Investigations Committee at the Yale University approved this study.

### Primary clinical outcome

Treatment effectiveness is modeled as the delta change scores of the SRS-parent total raw scores, that is, post minus pre, such that negative (positive) delta change scores indicate decrease (increase) in the core autism symptom severity. Treatment effectiveness was normally distributed, Shapiro–Wilk's *W*=0.96, *df*=20, *P*=0.45, and was uncorrelated with pretreatment SRS-parent total raw scores, *r*(18)=−0.35, *P*=0.13. To control for the passage of time, 11 of our participants were randomly assigned to a waitlist control group, and received treatment only after their 16-week waitlist period. The levels of ASD symptom severity did not significantly differ from the waitlist control baseline (−16 weeks; mean=91.27, s.d.=30.42) to the pretreatment baseline (0 weeks; mean=84.27, s.d.=24.06), Δ=−7.00, s.d. of Δ=15.79, *t*(10)=−1.47, *P*=0.17 (two-sided), 95% confidence interval of Δ=[−17.61, 3.61], Cohen's *d*_rm_^28^=0.24.

### Treatment approach

After the pretreatment scan was performed and the baseline clinical measures were taken, the participants received 16 weeks of PRT,^2, 12^ which is a naturalistic, behaviorally based treatment approach. PRT involves specific treatment components (child choice, child attending, clear opportunity, contingent reinforcement, natural reinforcement, reinforcement of attempts and interspersed maintenance/acquisition tasks) designed to increase the child's social motivation. In addition, PRT is highly naturalistic. In the context of the current study, the sessions were play-based, relying on materials such as craft supplies, balls, blocks and ‘play-doh'. For each child, the treatment included a total of 7 h of treatment per week. The sessions were held in the clinic as well as in the child's home. Five hours per week were direct intervention with the child, and 2 h per week consisted of parent-training sessions. The treatment targeted pivotal areas, including social initiation and responsivity, with the premise that improvements in these capacities should lead to more widespread and generalized improvements in multiple areas of development, representing core changes in social motivation. A more detailed description of PRT can be found in the original instruction manual^12^ and in an updated guide.^29^ Because the parent-training component is inherent in our treatment approach, parents were not blinded to the intervention. Overall, the sample reported here represents the provision of 2240 h of direct therapeutic intervention (1120 individual family visits), 20 one and a half hour scanning sessions, and 60 two-hour clinical evaluations, for a total of 1220 direct interactions (totaling 2390 h) with our 20 participating ASD families.

All the clinicians involved in the treatment were extensively trained in PRT. The faculty from the University of California Santa Barbara, the research institution where PRT was developed, trained the lead clinician (PV). The lead clinician sent two separate videotaped sessions (of different children) to the trainer to ensure maintenance of treatment fidelity. Both videos met the standard fidelity criteria. To ensure that the bachelors-level clinicians were correctly implementing PRT during their sessions, they met with the licensed (lead) clinician for 2 h per week. During these meetings, clinicians discussed the children's progress, current presentation and specific activities for the treatment sessions that would be motivating and foster skill development. In addition, the lead clinician observed sessions live and via videotape at least once weekly for each participant. Formal fidelity of implementation was assessed for two randomly coded treatment sessions for each subject. Two randomly selected 5 min segments per session were used for this fidelity assessment. The standard fidelity assessment published by the developers of the approach was used, and per convention, fidelity was defined as demonstrating the treatment components (child choice, child attending, clear opportunity, contingent reinforcement, natural reinforcement, reinforcement of attempts and interspersal of maintenance/acquisition tasks) in 80% of opportunities.^12, 29, 30^ The scoring was dichotomous; if the therapist demonstrated the component, a checkmark was used, and if not, a minus was used. All the therapists maintained the defined treatment fidelity across the duration of the study.

### Imaging task

We measured the pretreatment blood oxygen level dependent (BOLD) responses using a well-established biological motion fMRI task,^11, 31^ which was well tolerated by the young children with ASD in our study. We selected this paradigm to engage the brain regions involved in social perception, action observation, social cognition and social motivation. We reasoned these networks would be those most likely to relate to the targets of PRT. This same kind of task has been used with success in adults with and without ASD, as well as infant siblings of children with ASD, and toddlers with and without ASD. Thus, it represents a robust neuroimaging paradigm to measure the brain responses during social information processing across the lifespan in ASD. Before the treatment, the participants were scanned while viewing coherent and scrambled point-light displays of biological motion created from motion capture data. The coherent biological motion displays featured an adult male actor performing movements relevant to early childhood experiences, such as playing pat-a-cake,^19^ and contain 16 points corresponding to major joints. The scrambled motion animations were created by selecting all the 16 points from the biological motion displays and randomly plotting their trajectories on a black background. Thus, the coherent and scrambled displays contained the same local motion information, but only the coherent displays contained the configuration of a person.^20^ During the MRI scan, the stimuli were presented using E-Prime 2.0 software (Psychological Software Tools, Pittsburgh, PA, USA). Six coherent biological motion clips (BIO) and six SCRAM motion clips were presented (see Supplementary Figure 1) once each in an alternating-block design (time per block,~24 s). The experiment began with a 20 s fixation period and ended with a 16 s fixation period. The total duration was 328 s. The movies were presented without audio. The participants were asked to watch the videos and reminded to remain still and alert. Compliance with this request was facilitated via a mock scan before the actual scan and ensured by post-scan interview. All the children complied with this request. The imaging task and stimuli are available from the authors upon request.

### Imaging acquisition and processing

The scanning was performed on a Siemens MAGNETOM 3 Tesla Tim Trio scanner at the Yale Magnetic Resonance Research Center. For each participant, a structural MRI image series was acquired with a 32-channel head coil, a T1-weighted MPRAGE sequence, and the following parameters: 160 sagittal slices; repetition time (TR)=1900 ms; echo time (TE)=2.96 ms; flip angle=9° slice thickness=1.00 mm; voxel size=1 × 1 × 1 mm^3^; matrix=256 × 256; and field of view=256 × 256 mm^2^. Afterwards, BOLD T2*-weighted functional MRI images were acquired using the following parameters: 164 volumes; TR=2000 ms; TE=25 ms; flip angle=60° slice thickness=4.00 mm; voxel size=3.44 × 3.44 × 4.00 mm^3^; matrix=64 × 64; field of view=220 × 200 mm^2^; number of slices per volume=34; and interleaved acquisition.

The T1-weighted MPRAGE structural scan was segmented by SPM12 into gray matter, white matter and cerebrospinal fluid images. This method is highly accurate and has reduced bias relative to manual measurement.^32^

The fMRI data were processed using FSL^33^ v5.0.8 and the participant-level preprocessing steps followed a standardized processing stream—ICA-AROMA (ICA-based strategy for Automatic Removal of Motion Artifacts).^34^ This consisted of the following sequence: (a) motion correction using MCFLIRT; (b) interleaved slice timing correction; (c) BET brain extraction; (d) grand mean intensity normalization for the whole four-dimensional data set; (e) spatial smoothing with 5 mm full width at half maximum; (f) data de-noising with ICA-AROMA,^34^ which uses a robust set of theoretically motivated temporal and spatial features to remove motion-related spurious noise; (g) nuisance regression using time series for white matter and cerebrospinal fluid signal to remove residual, physiological noise; and finally (h) high-pass temporal filtering (100 s). The first 4 s were discarded to establish T1 equilibrium. Registration of the fMRI data was performed using both the subject's structural scan and then the Montreal Neurological Institute (MNI152) standard brain. Preprocessed data were then pre-whitened using FSL's FILM to remove time series autocorrelation.

To model the BIO and SCRAM conditions, the timing of the corresponding blocks was convolved with the default gamma function (phase=0 s, s.d.=3 s, mean lag=6 s) with temporal derivatives. The participant-level contrast of interest is BIO>SCRAM, which served as inputs for the subsequent mass univariate, whole-brain, group-level general linear model (GLM) analyses and multivariate pattern analyses. Sex was controlled for as a covariate of no interest across all group-level analyses. The main findings remained largely the same when sex was not controlled for in the analyses. The data sets during and/or analyzed during the current study are available from the corresponding author on reasonable request.

### Mass univariate group-level GLM analyses

We conducted mass univariate voxel-wise GLM analyses across the whole brain to identify clusters where pretreatment BOLD activation in the contrast of BIO>SCRAM predicted treatment effectiveness. The analyses were conducted using mixed-effects modeling with FSL's FLAME (FMRIB's Local Analysis of Mixed Effects) 1+2 inference algorithm, with a voxel-level threshold of *Z*>2.33, *P*<0.01 and corrected for multiple comparisons at a cluster-level threshold of *P*<0.05. Information about the surviving clusters was reported, including number of voxels in the cluster, the anatomical regions covered by the clusters based on the Automated Anatomical Labeling v2 (AAL2) atlas,^35^ the coordinates of the peak voxels within each of the anatomical regions and the Z-statistics associated with the peak voxels.

### Meta-analytical reverse inference

To understand the functional relevance of the surviving clusters, we performed a quantitative reverse inference using NeuroSynth (http://www.neurosynth.org/). The NeuroSynth data set v0.6 contains activation data for over 11 406 studies and feature information for over 3300 term-based features. The term-based features were derived from the abstracts of articles in the NeuroSynth database. For each feature, the database stores the whole-brain, reverse inference, meta-analysis map, *P*(Term | Activation), that is, the likelihood that a feature term is used in a study given the presence of reported activation.^36^ Each surviving cluster was decoded with NeuroSynth, which computed the voxel-wise Pearson correlation between the cluster image file and the meta-analytical image file associated with each of the 3300 feature terms. The top 10 psychological functional terms (for example, multisensory, reward) with the highest positive correlation were retained and reported, while we omitted non-functional terms, such as (but not limited to) those describing an anatomical region (for example, inferior temporal), a technique/method/task (for example, multivariate pattern), a population (for example, older adults), a disorder/disability/impairment (for example, cognitive impairment) or being relatively generic (for example, scale, weight, periods, emerged and so on).

### Multivariate pattern analyses

To guard against data over-fitting and to gain understanding of how different voxels in the network of the clusters derived from the mass univariate GLM analyses worked together in predicting treatment effectiveness, we utilized regression-based multivariate pattern analyses (MVPAs).^37^ In MVPA, the samples were divided into training and testing data sets, which constitute a *cross validation framework* in which the predictive model is first trained with the training set and then used to predict the regression labels of the sample in the testing set. This type of cross validation provides approximately unbiased estimates of effects, generalizable to new samples, helping to minimize the likelihood that the results over-fit the data. Moreover, in contrast to the mass univariate voxel-wise GLM analyses, MVPA draws on the multivariate information across many voxels comprising neural networks, which may capture how the voxels or regions work together to achieve complex functions. All these characteristics render MVPA well suited for establishing robust predictive biomarkers. MVPA has been applied to fMRI data to successfully predict treatment response or long-term outcome in a number of neuropsychiatric or neurocognitive disorders, such as depression,^38^ dyslexia,^39^ social anxiety disorder^40, 41^ and panic disorder.^42^

MVPAs were performed using the Pattern Recognition for Neuroimaging Toolbox^43^ (PRoNTo) v2.0 in Matlab and followed several steps. First, each participant's pretreatment Z-statistic BIO>SCRAM contrast image (up-sampled to the standard MNI152 space using trilinear interpolation) was inputted into the MVPA analyses. The surviving cluster(s) derived from the univariate analysis as a network was used as an analytical mask. Because our objective was to predict treatment effectiveness as a continuous variable, the delta change of ASD symptom severity was entered as the regression target. Second, PRoNTo computed a linear kernel (that is, dot product) between the voxel intensities within the mask for each pair of the input images, thereby generating a 20 × 20 similarity matrix, which served as the input feature set for the subsequent machine learning algorithm. Third, we used kernel ridge regression^44^ as the multivariate regression method. This is the dual-form formulation of ridge regression and solves regression problems with high dimensional data in a computationally efficient way. Cross validation was based on a leave-one-subject-out (LOSO) framework with mean-centered features across training images. We selected LOSO (which is equal to 20-fold cross validation with our sample) because a larger number of folds may reduce bias of the estimates, even at the cost of increasing variance of the estimates, and should provide more accurate estimates of neural predictability, especially when sample sizes are small. For each fold, one input image was left out and served as the testing set. The kernel ridge regression machines were trained to associate treatment effectiveness with the multivariate information in the remaining sample of 19 participants. The trained kernel ridge regression machines were then used to predict treatment effectiveness in the left-out image. This step was repeated for each of the 20 folds. Across all folds, predictive accuracy was calculated as the Pearson's correlation coefficient (*r*), coefficient of determination (*R*^2^), and normalized mean squared error (nMSE) between predicted and actual treatment effectiveness. Fourth, the significance of the prediction accuracy statistics was evaluated using a permutation test, consisting of 50 000 iterations. In each iteration, the regression targets were randomly permuted across all the participants and the cross-validation procedure was repeated. The *P*-values of *r*, *R*^2^ and nMSE were then calculated as the proportion of all permutations where *r*, *R*^2^ and nMSE were greater than (or less than, in the case of nMSE) or equal to the obtained *r*, *R*^2^ and nMSE, respectively.

## Results

### Primary clinical outcome

**Figure 1:**
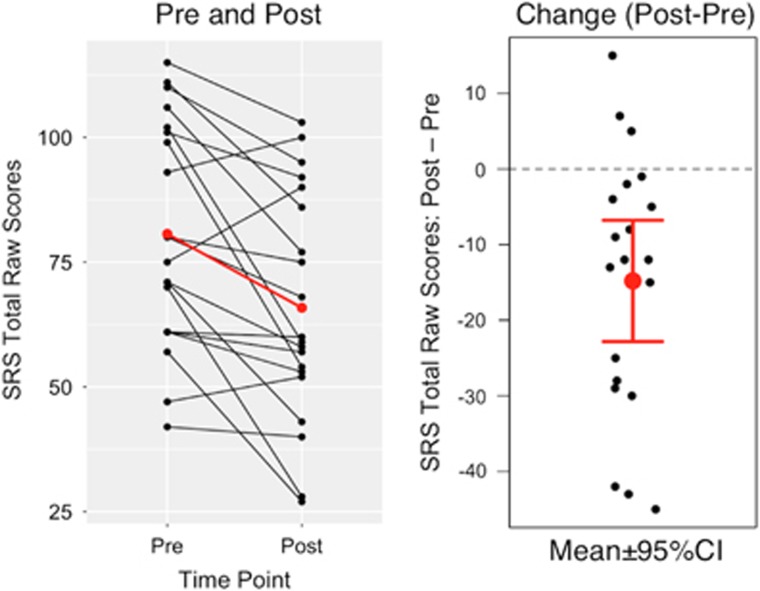
Treatment effectiveness quantified as the change in SRS total raw score. Left: the black lines indicate each child's change in core autism symptom severity from pretreatment to posttreatment; the red line is the group mean. Right: the mean and the 95% confidence interval (CI) of Δ, the change score (that is, post minus pre). SRS, Social Responsiveness Scale.

Comprehensive demographics and characterization information are provided in Table 1. As illustrated in Figure 1, PRT significantly reduced core ASD symptom severity in terms of parent-reported SRS total raw scores from pretreatment (mean=80.65, s.d.=22.53) to posttreatment (mean=65.85, s.d.=23.09), Δ=−14.80, s.d. of Δ=17.14, *t*(19)=−3.86, *P*=0.001 (two-tailed), 95% confidence interval of Δ=[−22.82, −6.78], Cohen's *d*_rm_^28^=0.65 (medium to large).

### Mass univariate GLM analyses

**Figure 2:**
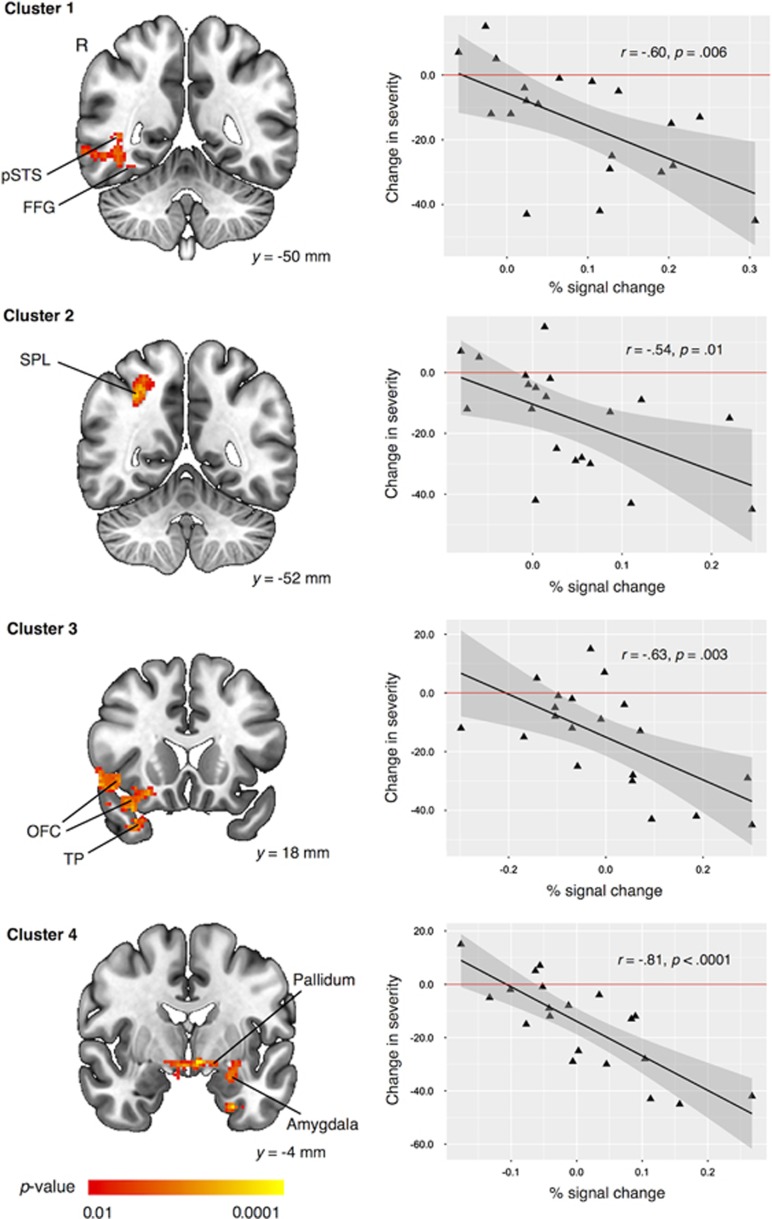
Prediction of treatment effectiveness using univariate general linear model (GLM). Four distinct brain regions, in which greater pretreatment BOLD activation (% signal change) in the contrast of biological vs scrambled motion was associated with greater treatment effectiveness. Scatterplot illustrating pretreatment BOLD activation and actual change in severity (that is, post minus pre), with a horizontal reference line at *y*=0 indicating no change from pretreatment to posttreatment (that is, post=pre). BOLD, blood oxygen level dependent; FFG, fusiform gyrus; OFC, orbital frontal cortex; pSTS, posterior superior temporal sulcus; R, right; SPL, superior parietal lobule; TP, temporal pole.

As illustrated in Figure 2, the whole-brain mass univariate GLM analyses of the pretreatment brain BOLD response to BIO vs SCRAM on the change in SRS total raw score from baseline to treatment end point revealed four distinct clusters of neuropredictive activities. Cluster 1 (359 voxels) contained a set of right-hemisphere brain areas involved in social perception: the fusiform gyrus, inferior temporal gyrus and middle temporal gyrus extending into the posterior superior temporal sulcus region.^9, 10^ Cluster 2 (403 voxels) included a set of right-hemisphere brain regions, part of the well-known social attention network^7^ and dorsal attention network,^45, 46, 47^ implicated in goal-directed (top-down) shifts in attention (for example, following gaze directions of others) and action observation^8^ and including (but not limited to) the inferior parietal gyrus and superior parietal lobule. Cluster 3 (534 voxels) included a set of right-hemisphere brain regions, well known for their role in the experience and regulation of emotion,^48^ as well as for coding the reward value of external stimuli:^49^ orbitofrontal cortex, ventrolateral prefrontal cortex, anterior insula and temporal pole. Finally, Cluster 4 (888 voxels) encompassed a set of left-hemisphere neuroanatomical structures commonly implicated in social memory and social motivation/social reward: putamen, pallidum, amygdala, hippocampus and ventral striatum.^6, 50^ Figure 2 also demonstrates the form of the neuropredictive relationship with a scatterplot of the change in core ASD symptom severity (*y* axis) vs pretreatment BIO>SCRAM activity (*x* axis) for each of the four clusters. As can be seen, greater levels of pretreatment activation in these circuits were negatively correlated with changes in severity brought about by PRT, such that greater pretreatment activation was associated with greater reduction in severity. There was no region that showed positive correlations between pretreatment activation and changes in severity. Supplementary Table 1 lists the peak significance, spatial extent and anatomical locations encompassed by each predictive cluster.

We conducted a NeuroSynth-based (http://neurosynth.org) reverse inference analysis to further interpret the possible functions of the neuropredictive clusters. As illustrated in Supplementary Table 2, Cluster 1 correlates with multisensory and cross modal perception, response selection, object perception and motion perception. Cluster 2 correlates with numerical processing (which is one of functions of the intraparietal cortex,^51^ besides social attention) and visuospatial attention. Cluster 3 correlates with constructs including response inhibition and emotion regulation. Finally, Cluster 4 correlates with the constructs of reward and motivation (for example, sexual, reward, unpleasant, pleasant, motivation). The image files from this analysis are available at http://neurovault.org/collections/1551/ so that interested readers may independently decode the image files with NeuroSynth through links within the NeuroVault website.

### Multivariate pattern analyses with cross validation

**Figure 3:**
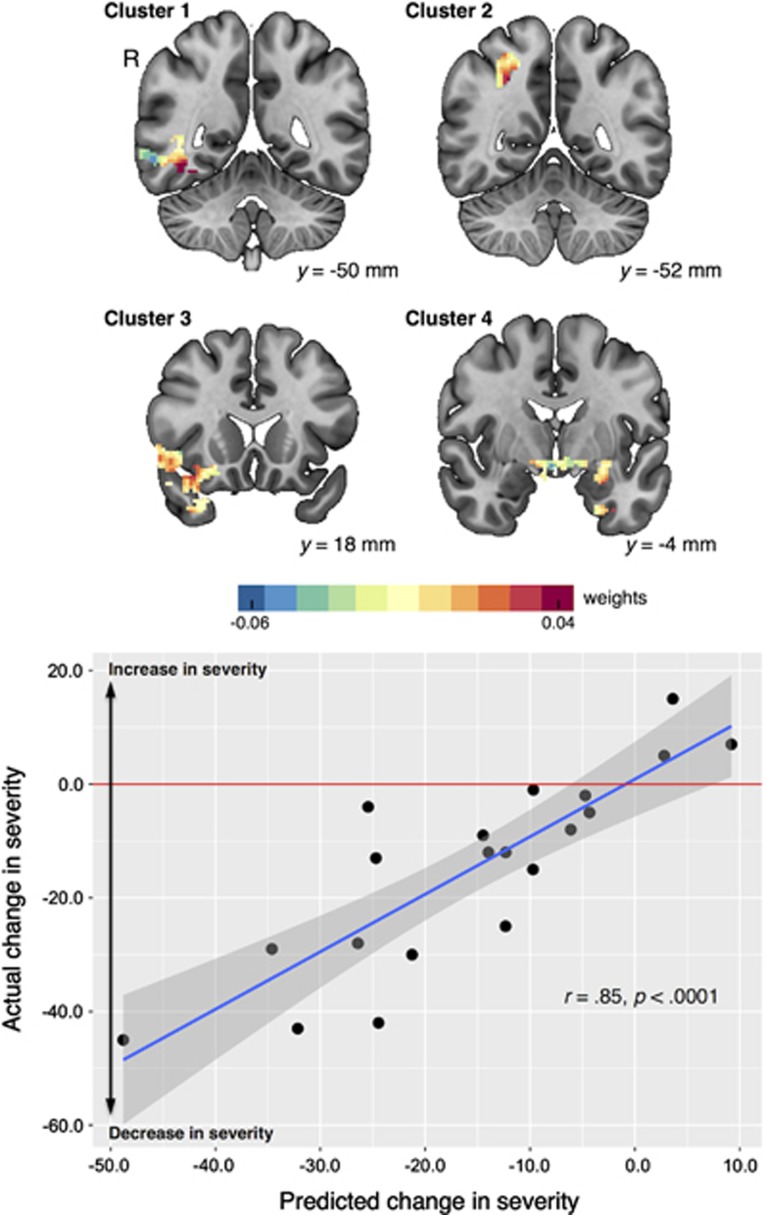
Predictive accuracy of the univariate neuropredictive clusters, as estimated by MVPA with cross validation. Top: weight map showing the relative weights derived from the multivariate modeling of pretreatment response to biological motion that contributed to the prediction of change in severity (that is, post minus pre) at representative slices (MNI152 mm space). Bottom: scatterplot illustrating actual and predicted treatment effectiveness, with a horizontal reference line at *y*=0 indicating no change from pretreatment to posttreatment (that is, post=pre). Cross validation was based on a leave-one-subject-out framework. MNI, Montreal Neurological Institute; MVPA, multivariate pattern analysis; R, right.

To guard against the possibility of data over-fitting in the mass univariate analysis, and to gain an understanding of how the voxels comprising the univariate clusters work together in predicting treatment effectiveness, we applied regression-based MVPA of pretreatment BOLD responses to the contrast of BIO>SCRAM with LOSO cross validation in the voxels comprising the four univariate clusters. As shown in Table 2, the neuropredictive network consisting of the four clusters survived cross validation—the multivariate pattern information from this brain network significantly predicted treatment outcome (*r*=0.85, *P*<0.0001; *R*^2^=0.72, *P*=0.0001; *nMSE*=1.33, *P*<0.0001). Figure 3 (top) shows the weight map (that is, model parameters) in the representative slices of this network derived from the multivariate modeling of pretreatment images predicting treatment response. Figure 3 (bottom) shows the scatter plot of actual vs predicted treatment response. Each of the points in this plot was derived from a separate training set, whereas for a new unseen child (testing set), the remaining participants' data were used as the training set. Thus, the correlation is not a standard correlation derived from a single set of participants. Rather, each point reflects different combinations of training and testing sets.

We also conducted MVPA analyses with a comparison/control region of interest that we did not expect to be predictive of treatment outcome. The inferior occipital gyrus was selected because (i) it is roughly the same size as the neuropredictive network, yet it does not overlap with the neuropredictive network, (ii) it responds strongly to a range of visual stimuli including the SCRAM and BIO stimuli used here and (iii) it was shown to be neuropredictive of treatment effectiveness in a markedly different neuropsychiatric condition, social anxiety disorder.^1, 52^ Furthermore, we conducted MVPA with the whole brain (including the neuropredictive network) to evaluate the specificity of our findings to the network of these four univariate clusters. As shown in Table 2, neither the comparison region of interest nor the whole-brain analysis was predictive of treatment outcome (*P*-values >0.05).

### Demographic and behavioral findings

To evaluate whether fMRI provides unique information concerning the prediction of response to PRT, we examined how a host of demographic and pretreatment clinical behavioral measures predict treatment outcome. We ran correlation analyses between each of the measures listed in Table 1 and the delta changes in SRS total raw scores. No measure showed a significant correlation, *P*-values >0.05.

## Discussion

Among young, cognitively able boys and girls with ASD, we discovered a brain network in which the pretreatment brain activities engaged during biological motion viewing predict treatment response to an evidence-based behavioral intervention. Specifically, the network includes key brain regions supporting social information processing (the superior temporal sulcus region, fusiform gyrus, superior parietal lobule) and social motivation (orbitofrontal cortex, putamen, ventral striatum). Critically, the results were supported by MVPA, which utilized a standard cross validation framework, suggesting that the patterns of brain activities across these brain regions may serve as robust predictive biomarkers, generalizable to new, unseen participants.

To our knowledge, the current findings provide the first clear evidence of a neuroimaging-informed stratification/predictive biomarker in ASD. Our findings move the field toward the goal of targeted, personalized treatment for individuals with ASD. The knowledge gained can be utilized in future work to tailor individualized treatment, refine PRT and develop novel interventions. This study adds to the understanding of the pretreatment neural underpinnings of successful behavioral response to PRT. In the future, our results may drive the construction of algorithms to predict which, among several treatments, is most likely to benefit a given person. In addition, PRT is a multi-component treatment; hence future studies might use dismantling designs to isolate treatment components and their association with the neuropredictive targets identified here. This line of work could inform the development of treatment strategies that would target specific patterns of neural strengths and vulnerabilities within a given patient—consistent with the priority of creating individually tailored interventions, customized to the characteristics of a given person.

The predictive biomarkers identified in this paper can be interpreted as the pretreatment neurobiological readiness to respond to a specific treatment, PRT. It should be noted that the brain regions where activity before treatment correlated with SRS scores before treatment (see Supplementary Table 3) did not overlap with the neuropredictive network described here, which indicates that the neuropredictive network is specific to change in severity in young children with ASD. As such, our findings offer the hope that pre- or concurrent-treatments (whether pharmacological, direct stimulation, neurofeedback, or behaviorally based) that improve the functioning of the neuropredictive markers identified here, may increase the effectiveness of evidenced-based behavioral treatments for core deficits in children with ASD. On the other hand, our findings are also particularly important for those children who would otherwise be the least likely to benefit from these expensive and time-consuming forms of treatment. For example, in a randomized, double-blind, cross-over functional fMRI study,^53^ we reported that intranasal oxytocin administered to children with ASD increases activity during social vs nonsocial judgments in several of the same brain regions identified as predictive in the present study (for example, amygdala, orbitofrontal cortex, superior temporal sulcus region and ventral striatum). These findings, coupled with those in the current report, raise the provocative hypothesis that the administration of intranasal oxytocin, by priming key neural circuits for social motivation and social perception, may serve to enhance the effectiveness of interventions like PRT in the very children who might be less biologically ready to respond.

### Limitations

There are several limitations that should be considered regarding this research. First, while our research is the first to identity neuropredictive biomarkers in the field of ASD and we did not have sufficient information regarding established effect sizes that would allow us to pre-determine the required sample size, the overall sample size (*n*=20) is relatively small, although a power analysis utilizing G*Power^54^ indicated that it is sufficiently powered (*β*=0.80) to detect a large size of effect^55^ (|*r*|⩾0.50, one-sided; in this research, *r* refers to the correlation between pretreatment brain activation level and treatment effectiveness). Future research should use a larger sample to detect small-to-medium sizes of effect. Second, the primary clinical outcome is the delta change score of the parent-reported SRS total raw score, and given that the parent training is inherent in the treatment approach, the parents were not (and could not be) blinded to the intervention. As such, there is a need for future research to include measures that are more objective and/or filled out by blinded clinician(s), which would provide a more comprehensive picture of treatment outcome, although the parent-reported SRS total raw score is one of a very few measures that could provide continuous quantification of symptom severity in ASD in naturalistic settings. Third, our neuropredictive findings were limited to one single treatment-only group in a pretest–posttest design, and future work should conduct randomized controlled trials to further establish these findings. Finally, although MVPA with LOSO cross validation provides supporting evidence that our univariate biomarkers may generalize to new, unseen samples, the results are nonetheless limited to the current data and thus the generalizability should be further tested in an independent sample beyond the current data.

## Conclusions

Early childhood provides an important window of opportunity for intervention in ASD. The promise of targeted, individualized, precision treatment for core deficits in ASD depends on sensitive, objective biomarkers that can predict how individual young children with ASD will respond to specific treatment(s). For the first time in the field of ASD, we provide evidence that neural signatures in brain circuits implicated in social information processing and social motivation/reward can predict treatment effectiveness at the individual level in young boys and girls with ASD. The results open a new avenue for important future research and should greatly accelerate progress toward more precise and effective treatments for core deficits in ASD.

## Figures and Tables

**Table 1:** Participants demographics and pretreatment characteristics

*Variable*	*Mean (s.d.)*
Pretreatment age (years)	5.90 (1.07)
Gender, male (0=f, 1=m)	0.65 (0.49)
General conceptual ability (IQ)	103.45 (17.03)
Handedness, right (1=right, 0=ambi., −1=left)	0.70 (0.66)
Pretreatment ADOS calibrated severity score	7.65 (2.11)
Pretreatment SRS-parent total raw score	80.65 (22.53)
Pretreatment CELF core standard score	90.40 (23.88)
	
Pretreatment Vineland-II communication	
Receptive	39.60 (19.09)
Expressive	47.45 (13.01)
Written	75.45 (13.35)
	
Pretreatment Vineland-II daily living skills	
Personal	49.50 (13.13)
Domestic	54.05 (22.92)
Community	61.80 (18.26)
	
Pretreatment Vineland-II socialization	
Interpersonal relationships	40.35 (11.99)
Play and leisure time	46.25 (16.93)
Coping skills	41.30 (17.87)

Pretreatment scan, head motion (mean absolute, mm)	1.32 (1.34)
Pretreatment scan, head motion (mean relative, mm)	0.44 (0.44)

Abbreviations: ADOS, Autism Diagnostic Observation Schedule; ambi., ambidextrous; CELF, Clinical Evaluation of Language Fundamentals; f, female; IQ, intelligence quotient; m, male; SRS, Social Responsiveness Scale; Vineland-II, Vineland Adaptive Behavior Scales-Second Edition.

Vineland-II scores were age equivalents in months. Treatment outcome was the change score of SRS-parent total row score, that is, post minus pre. Number of participants: gender=7 f/13 m; handedness=16 right/2 ambi./2 left.

**Table 2:** Predictive accuracy of the univariate neuropredictive clusters, as estimated by MVPA with cross validation

*Mask*	*N*_voxels_	*r*	*P*_(*r*)_	*R*^*2*^	*P*_*(*R^*2*^__*)*_	*nMSE*	*P*_(*nMSE*)_
Univariate neuropredictive network	**2184**	**0.85**	**<0.0001**	**0.72**	**0.0001**	**1.33**	**<0.0001**
Inferior occipital gyrus	1930	−0.14	0.62	0.02	0.66	6.89	0.39
Whole brain	228 453	0.16	0.17	0.02	0.67	4.63	0.15

Abbreviation: MVPA, multivariate pattern analysis.

Prediction accuracy was indicated by Pearson's correlation coefficient (*r*), coefficient of determination (*R*^2^) and normalized mean squared error (nMSE) between predicted and actual treatment effectiveness. Significance (*P*-value) was determined with a random permutation test (50 000 iterations). Significant regions and statistics were displayed in bold. Cross validation was based on a leave-one-subject-out framework.
