# Supplementary material for: Brain responses to biological motion predict treatment outcome in young children with autism
Source: Transl Psychiatry. 2016 Nov 15;6(11):e948–. doi: 10.1038/tp.2016.213 (PMC5314125; doi:10.1038/tp.2016.213)
Supplement: Supplementary Information [file tp2016213x1.docx]

**Supplementary Information for**

Brain Responses to Biological Motion Predict Treatment Outcome
in Young Children with Autism

Daniel Y.-J. Yang, Kevin A. Pelphrey, Denis G. Sukhodolsky, Michael J. Crowley, Eran Dayan, Nicha Dvornek, Archana Venkataraman, James Duncan, Lawrence Staib, Pamela Ventola

Correspondence to: daniel.yj.yang@yale.edu or pamela.ventola@yale.edu

This PDF file includes:

- Supplementary Tables 1-3
- Supplementary Figure 1

**Supplementary Table 1** Prediction of treatment effectiveness using univariate GLM

|  |  | Local Maxima | | | | | |
| --- | --- | --- | --- | --- | --- | --- | --- |
| Cluster | *N*_voxels_ |  | Anatomical Region | *x* | *y* | *z* | *Z*_peak_ |
| 1 | 359 | R | Fusiform gyrus | 32 | -48 | -14 | 2.92 |
|  |  | R | Inferior temporal gyrus | 62 | -58 | -8 | 3.93 |
|  |  | R | Middle temporal gyrus | 58 | -60 | 0 | 3.38 |
| 2 | 403 | R | Angular gyrus | 30 | -52 | 44 | 3.40 |
|  |  | R | Middle occipital gyrus | 32 | -66 | 34 | 2.97 |
|  |  | R | Inferior parietal gyrus | 32 | -50 | 42 | 3.39 |
|  |  | R | Superior parietal gyrus | 28 | -50 | 48 | 3.45 |
|  |  | R | Supramarginal gyrus | 36 | -40 | 44 | 2.87 |
| 3 | 534 | R | Inferior frontal gyrus, opercular part | 54 | 18 | -2 | 2.95 |
|  |  | R | Inferior frontal gyrus, orbital part | 52 | 18 | -10 | 3.20 |
|  |  | R | Inferior frontal gyrus, triangular part | 54 | 20 | 0 | 3.10 |
|  |  | R | Insula | 42 | 16 | -6 | 3.16 |
|  |  | R | Posterior orbital gyrus | 38 | 20 | -20 | 3.41 |
|  |  | R | Olfactory cortex | 24 | 14 | -16 | 3.42 |
|  |  | R | Lenticular nucleus, Putamen | 32 | 6 | -6 | 3.34 |
|  |  | R | Gyrus rectus | 22 | 12 | -16 | 3.02 |
|  |  | R | Temporal pole: middle temporal gyrus | 32 | 18 | -36 | 3.16 |
|  |  | R | Temporal pole: superior temporal gyrus | 54 | 16 | -10 | 3.37 |
| 4 | 888 | L | Amygdala | -26 | -4 | -18 | 3.01 |
|  |  | L | Fusiform gyrus | -28 | -8 | -36 | 4.07 |
|  |  | L | Hippocampus | -28 | -12 | -24 | 2.99 |
|  |  | L | Lenticular nucleus, Pallidum | -20 | 4 | 0 | 3.11 |
|  |  | L | Parahippocampal gyrus | -30 | -12 | -28 | 4.00 |
|  |  | L | Lenticular nucleus, Putamen | -22 | 4 | 0 | 3.09 |
|  |  | L | Inferior temporal gyrus | -36 | -10 | -36 | 3.43 |
|  |  | L | Thalamus | -10 | -10 | -2 | 3.10 |

*Note.* The coordinates are in MNI152 space, mm. Results were thresholded at *Z*>2.33 (*p*<.01) and corrected for multiple comparisons at the cluster level (*p*<.05). Sex was controlled for as a covariate of no interest in this additional analysis. R, Right; L, Left.

**Supplementary Table 2** Reverse inference analysis of the neuropredictive clusters using NeuroSynth

| Cluster | Top 10 NeuroSynth-decoded feature terms |
| --- | --- |
| 1 | Multisensory (.09), cross modal (.05), complex (.05), visual (.04), perception (.04), modality (.03), switch (.03), response selection (.03), objects (.03), motion (.02) |
| 2 | Numerical (.18), arithmetic (.13), attentional (.12), visuospatial (.11), spatial (.08), working memory (.07), attention (.05), visual attention (.04), attention network (.04), execution (.04) |
| 3 | Psychological (.14), game (.07), inhibition (.06), inhibitory control (.04), mood (.04), response inhibition (.04), regulating (.03), inhibiting (.03), choice (.03), emotional information (.03) |
| 4 | Sexual (.13), arousal (.13), negative emotional (.11), unpleasant (.10), motivation (.10), valence (.10), emotional stimuli (.10), reward (.09), pleasant (.08), food (.08) |

*Note*. Numbers within the parentheses are correlation coefficients between the surviving clusters and the meta-analysis maps of the feature terms in NeuroSynth.

**Supplementary Table 3** Peaks of regions in which pretreatment severity (SRS total raw scores) was negatively correlated with pretreatment responses to biological vs. scrambled motion (331 voxels)

|  | Anatomical Region | *x* | *y* | *z* | *Z*_peak_ |
| --- | --- | --- | --- | --- | --- |
| R | Caudate | 18 | -10 | 20 | 3.02 |
| R | Precentral gyrus | 38 | -16 | 38 | 2.51 |
| R | Thalamus | 16 | -10 | 16 | 2.89 |

*Note.* The coordinates are in MNI152 space, mm. Results were thresholded at *Z*>2.33 (*p*<.01) and corrected for multiple comparisons at the cluster level (*p*<.05). Sex was controlled for as a covariate of no interest in this additional analysis. R, Right.


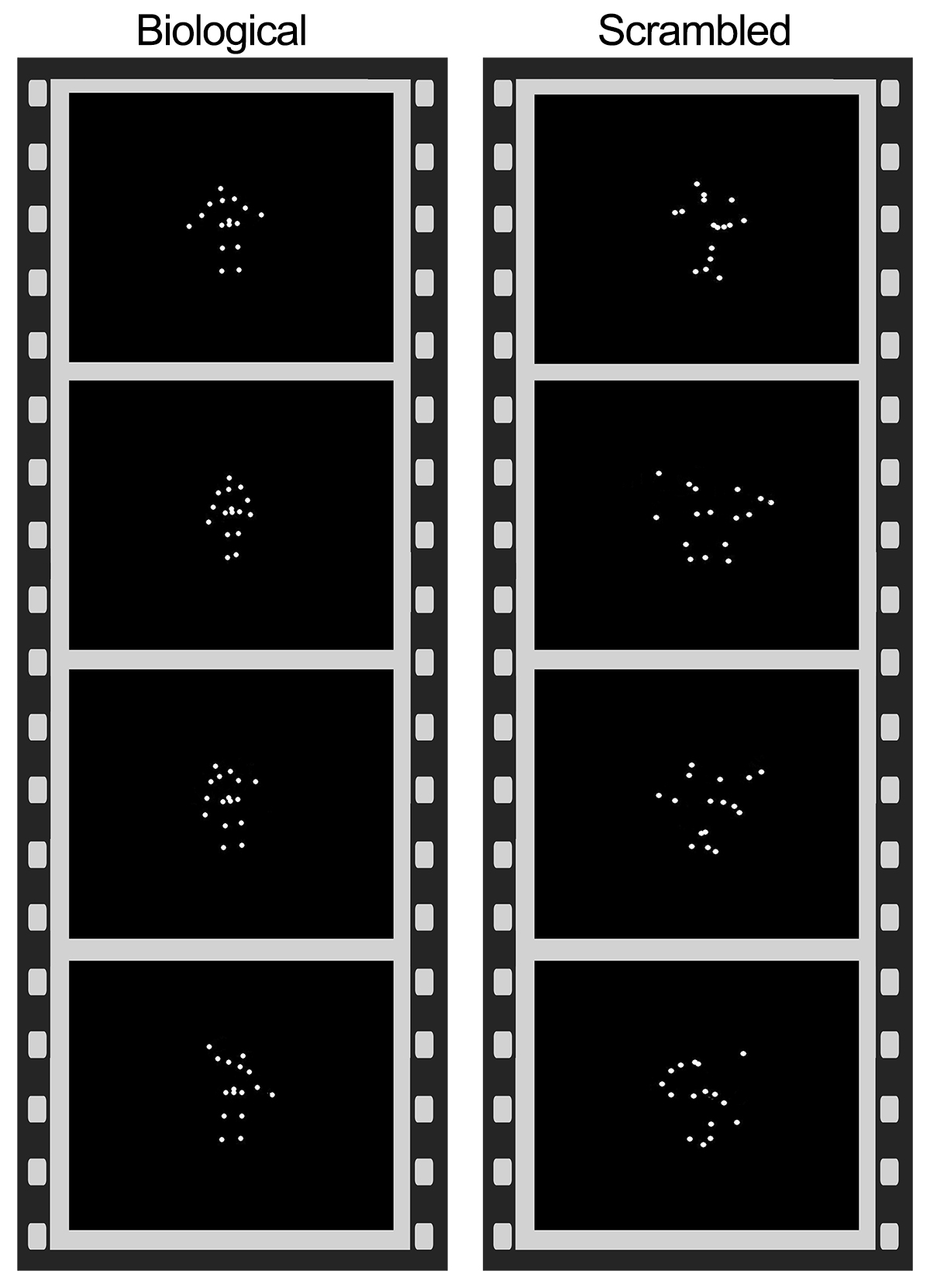


**Supplementary Figure 1. fMRI stimuli: Biological and scrambled motion**. The paradigm and stimuli are available from the authors upon reasonable request.
